# A review of *Elocomosta* Hansen with a description of a new species with reduced eyes from China (Coleoptera, Hydrophilidae, Sphaeridiinae)

**DOI:** 10.3897/zookeys.607.7142

**Published:** 2016-07-27

**Authors:** Renchao Lin, Fenglong Jia, Martin Fikáček

**Affiliations:** 1 Institute of Entomology, Sun Yat-sen University, Guangzhou, 510275, Guangdong, China; 2 Department of Entomology, National Museum in Prague, Cirkusová 1740, CZ-19100 Praha 9, Czech Republic; 3 Department of Zoology, Faculty of Science, Charles University in Prague, Viničná 7, CZ-128 44 Praha 2, Czech Republic

**Keywords:** China, Coelostomatini, *Elocomosta*, eye reduction, new species

## Abstract

A new species of the genus *Elocomosta* Hansen, 1989 (Coleoptera: Hydrophilidae: Sphaeridiinae: Coelostomatini), *Elocomosta lilizheni* **sp. n**., is described from Guangxi Province, China. It is compared in detail with the only other known species of the genus, *Elocomosta nigra* Hansen, 1989 from Borneo, and the genus is diagnosed from the remaining coelostomatine genera. The new species is unusual among Hydrophilidae by having extremely reduced eyes.

## Introduction

The tribe Coelostomatini is one of the largest groups of hydrophilid beetles, represented by 17 genera containing more than 200 described species (Short and Fikáček 2011, 2013). Only two of these genera are species-rich: the aquatic genus *Coelostoma* Brullé, 1835 with slightly more than 100 described species (e.g., Short and Fikáček 2011, Jia et al. 2014), and the terrestrial genus *Dactylosternum* Wollaston, 1854 with *ca.* 70 described species (Short and Fikáček 2011). The remaining coelostomatine genera contain far fewer species, usually characterized by peculiarities of ventral morphology. In some cases, these peculiarities are likely related to the specific biology as in the case of *Lachnodacnum* Orchymont, 1937 inhabiting bromeliads in Brazil (Clarkson et al. 2014) or *Coeloctenus* Balfour-Browne, 1939 inhabiting the tidal zone of Lake Victoria (Balfour-Browne 1939). In some genera (e.g., *Phaenonotum* Sharp, 1882 and *Phaenostoma* Orchymont, 1937), new species continue to be discovered by recent faunal inventories (Gustafson and Short 2010, Deler-Hernández et al. 2013), indicating that they are not as species-poor as they currently may appear.

*Elocomosta* Hansen, 1989 was erected for a single aberrant species of Coelostomatini from the Malaysian province of Sarawak on the island of Borneo, at that time placed in the tribe Sphaeridiini. The genus was transferred to Coelostomatini by Hansen (1991) and remained virtually unknown, with only few series of specimens collected after the description, all from Borneo and all belonging to the type species. It was hence a big surprise when an aberrant coelostomatine species with reduced eyes collected in the Guangxi province of south China was identified as *Elocomosta* using the key by Hansen (1991). It encouraged a detailed comparison of the Chinese species with the type species of *Elocomosta*, which is summarized here.

## Material and methods

Two specimens of *Elocomosta nigra* and 12 specimens of the new species have been examined for this study. The holotype and several of the paratypes of the new species were dissected. After eight hours in 10% KOH at room temperature, male genitalia were mounted in glycerol on a piece of transparent plastic pinned below each specimen. Morphological characters and aedeagi were examined with the use of Leica S8APO compound microscope. The external morphology was examined using the Hitachi S-3700N environmental electron microscope at the Department of Paleontology, National Museum in Prague. Habitus photographs were taken using Canon D-550 digital camera with attached Canon MP-E65mm f/2.8 1–5 × macro lens, and subsequently adapted in Adobe Photoshop CS2. Aedeagus photographs were taken with an Axioskop 40, and then stacked using Auto-Montage software. Morphological terminology largely follows Clarkson et al. (2014), the higher-level taxonomic nomenclature follows Hansen (1999) and Short and Fikáček (2011, 2013).

Examined specimens are deposited in the following collections:

**NMPC** National Museum, Prague, Czech Republic;

**SHNU** Shanghai Normal University, China;

**SYSU** Sun Yat-sen University, Guangzhou, China.

## Taxonomy

### Elocomosta

Taxon classificationAnimaliaColeopteraHydrophilidae

Hansen, 1989

Elocomosta Hansen, 1989: 254.

#### Type species.

*Elocomosta nigra* Hansen, 1989 (by original designation).

#### Diagnosis.

The genus may be easily diagnosed from other coelostomatine genera by the combination of the following characters: antenna with thin, loosely segmented club, maxillary palpomere 2 without apparent distal widening, elytron with ten series of punctures (usually not very apparent among interval punctures) but without sutural stria, prosternum rather long in front of procoxae, mesoventrite with subpentagonal to circular plate with marginal bead, without anepisternal sutures and anteromedian pit, metaventrite very short but with long and wide metaventral process abutting posterior margin of mesoventral plate, abdominal ventrite 1 without median carina and abdominal ventrite 5 without apical emargination and/or group of stout setae.

*Elocomosta* is easy to distinguish from other Oriental coelostomatine genera by the morphology of the mesoventral plate, which is more or less flat and with a bead along the whole margin. In this it differs from *Coelostoma* Brullé, *Coelofletium* Orchymont, and *Dactylosternum* Wollaston, in which the mesoventral plate is keel- or roof-like and generally of the arrow-head-shape morphology. *Rhachiostethus* Hansen has the mesoventral plate flat, but it is tightly fused to the elevated metaventral keel and lacks the marginal bead. *Dactylostethus* Orchymont bears the mesoventral plate which is very similar to that of *Elocomosta* in the shape and in the presence of the marginal bead, but can be easily distinguished from *Elocomosta* by an extremely short prosternum, elytra without any traces of puncture series, abdominal ventrite 1 with median carina, more compact antennal club and mesoventrite with the anteromedian pit-like depression.

#### Redescription.

Body widely oval, moderately convex. General coloration of dorsum blackish. Body length 2.3–2.9 mm.

*Head* situated in a deep anterior emargination of pronotum. Frontoclypeal suture indistinct, only partially developed; preocular portion of frons (between eye and frontoclypeal suture) rather wide. Eyes well developed and strongly constricted laterally, or largely reduced. Anterior margin of clypeus without marginal bead. Labrum weakly sclerotized, largely membranous, narrowly to largely exposed in front of clypeus. Gula very narrow, posterior tentorial pits small and inconspicuous, situated close to each other. Postocular ridges strongly developed, reaching behind cardines. Maxilla with transverse cardo and triangular basistipes, basistipes with sparsely arranged long setae; galea large, partly membranous, with pubescence arranged in series; maxillary palpus with four palpomeres, basal palpomere minute, palpomeres 2–3 only indistinctly widened apically, palpomere 4 cylindrical, palpomere 2 the longest, *ca.* 1.5 × longer than palpomeres 3 and 4, palpomeres 3-4 subequal in length. Mentum transverse, slightly widening from base anteriad, lateral sides with series of fine setae, anterior margin bisinuate and with transverse subanterior ridge, surface with sparsely arranged long setae; labial palpus with three palpomeres, palpomere 1 minute, palpomere 2 the longest, *ca.* 1.3 × as long as palpomere 3, bearing subapical fringe of setae, palpomere 2 cylindrical, with subapical setae. Antenna with nine antennomeres; scapus *ca.* twice as long as pedicellus, antennomere 3 thin and *ca.* as long as antennomeres 4–5 combined, antennomeres 4–5 slightly widening distally, cupule (antennomere 6) rather wide distally much wider than antennomere 7, antennomeres 7–9 pubescent, forming a very thin loosely segmented club.

*Prothorax*. Pronotum transverse, deeply excised on anterior margin, strongly widened posterially; anterior and lateral margin with complete marginal bead, posterior margin without marginal bead; posterolateral corners rectangular to acute, posterior margin nearly straight to slightly concave; sublateral portions of pronotum with minute but distinctly developed trichobothria. Prosternum in front of procoxae rather wide, medially flat, without longitudinal ridge, only with a weak transverse impression along anterior margin; prosternal process hidden below procoxae, hence posterior margin of exposed portion of prosternum widely triangular; anterior margin angulate medially, with fine marginal bead, posterior margin finely beaded; concealed portion of prosternal process slightly widened posterior of procoxae. Procoxal cavities contiguous medially, open posteriorly, anterolateral aperture of procoxae very narrowly open to completely closed. Hypomeron with wide lateral bare portion, divided by a very fine line from median pubescent portion; anteromesal portion with a rather indistinct “antennal groove” defined by a weak ridge.

*Pterothorax*. Scutellar shield rather small, in shape of equilateral triangle. Elytron with ten longitudinal series of punctures but without sutural stria; scutellar stria not developed. Trichobothria minute but present on alternate elytral intervals; epipleuron wide and horizontal throughout, with wide external bare portion. Punctures of elytral intervals with a characteristic structure of several concentric ridges. Mesothorax with strongly elevate mesoventral plate of elongate subpentagonal to circular shape, margins of the plate with distinct wide marginal bead; posterior margin of the plate widely abutting metaventral process; anepisternal sutures reduced, not visible, anteromedian pit absent. Mesanepimeron rather narrow but long, completely closing mesocoxal cavity laterally. Metaventrite transverse, behind mesocoxae very short, shorter than the length of mesoventrite; median portion with wide and long metaventral process, *ca.* as long as metaventrite between meso- and metacoxae. Posterior margin of mesocoxae with a postcoxal ridge which does not continue to metaventral process. Median portion of metaventrite slightly elevated, with sparse pubescence similar to lateral portions of metaventrite, without surface microsculpture. Posterior half of metaventrite with fine longitudinal median carina. Metanepisternum narrow, *ca.* of the same width throughout, sparsely pubescent, with wide and long posterolateral process contacting abdomen. Apterous species.

*Legs* rather short, tips of femora not overlapping body outline. Procoxa subglobular, sparsely pubescent; profemur with sharply defined tibial groove; protibia cylindrical, with strong apical spines and a sparse series of lateral spines; protarsus densely pubescent ventrally, protarsomere 1 longest, *ca.* 2 × longer than each subsequent tarsomere. Mesocoxae rather widely divided from each other by metaventral process, transverse; metacoxae transverse, contiguous medially; meso- and metafemora rather wide basally, with sharply defined tibial grooves in distal half, ventral surface without hydrophobic pubescence, only with sparsely arranged setae; meso- and metatibiae slightly bent outwards, slightly widened distally, with short but stout spines distally and along lateral and mesal faces; meso- and metatarsomere 1 the longest, ca. 1.5–2.0 × as long as tarsomere 2; ventral face with dense pubescence, dorsal face with few isolated long setae.

*Abdomen* with 5 ventrites, all ventrites without median carina, abdominal apex without emargination or series/group of enlarged setae. All ventrites with dense hydrofuge pubescence.

**Figures 1–8.**
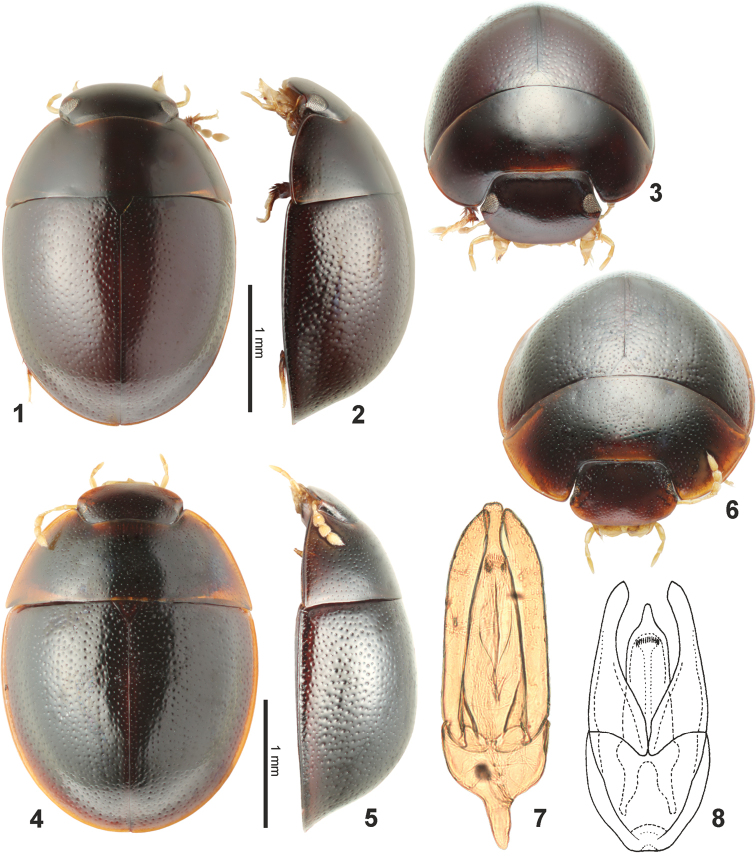
General habitus and genitalia of *Elocomosta* species. **1–3** *Elocomosta nigra* Hansen, 1989, paratype (**1** dorsal **2** lateral **3** frontal view) **4–6** *Elocomosta lilizheni* sp. n., paratype (**4** dorsal **5** lateral **6** frontal view) **7–8** aedeagus (**7** *Elocomosta lilizheni* sp. n., holotype **8** *Elocomosta nigra*, adapted from Hansen (1989).

**Table 1.** Differences between two known species of *Elocomosta* Hansen.

	*Elocomosta nigra* Hansen	*Elocomosta lilizheni* sp. n.
Coloration	Uniformly black	Black with orange margins of elytra and pronotum
Eyes	Well developed (head hence transverse)	Nearly completely reduced (head hence rather narrow)
Head punctation	Fine and sparse	Coarse and denser
Anterior margin of clypeus	Without microsculpture	With distinct transverse microsculpture
Labrum	Nearly completely concealed by clypeus	Partly exposed in front of clypeus
Punctation of pronotum	Much finer than on elytra	Almost as coarse as on elytra
Posterolateral corner of pronotum	Rectangular	Sharp (projecting more posteriorly)
Profemur	Basal ¾ covered by dense pubescence	Whole surface with sparse pubescence only
Elytron and epipleuron	Not explanate laterally, epipleuron moderately wide	Explanate laterally, epipleuron very wide
Mesoventral plate	Circular, almost as long as wide	Subpentagonal, 1.2 × longer than wide
Metaventral process	Narrowing anteriorly	Nearly parallel-sided
Metanepisternum	With apical lateral tooth	Without apical lateral tooth
Phallobase of the aedeagus	Long, without manubrium	Short, with long manubrium
Median lobe of the aedeagus	Reaching tips of parameres, blunt at apex	Not reaching tips of parameres, acute at apex

### Elocomosta lilizheni sp. n.

Taxon classificationAnimaliaColeopteraHydrophilidae

http://zoobank.org/A773D75E-007A-47E9-AB88-2D895CF33F8E

Figs 4–7 , 10 , 12 , 14 , 17 , 19–20 , 23 , 25 , 27 , 29–31 , 33

#### Type material.

Holotype: male (SYSU): China, Guangxi, Jinxiu County, Yinshan Conservation Station, 24°10'01"N, 110°14'38"E, beech forest, mixed leaf litter, sifted, 1200 m, 11.vii.2014, Peng, Song, Yu & Yan leg. Paratypes (11 spec., SYSU, SHNU, NMPC): 7 spec.: same information as the holotype; 2 spec.: China, Guangxi, Jinxiu County, 7 km, 24°09'07"N, 110°12'29"E, beech forest, mixed leaf litter, humus, sifted, 1300 m, 16.vii.2014, Peng, Song, Yu & Yan leg.; 2 spec.: China, Guangxi, Jinxiu County, Laoshan Forest Farm, 24°07'02"N, 110°11'51"E, on dead wood with Polypores, caught, 950 m, 26.vii.2014, Peng, Song, Yu & Yan leg.

#### Diagnosis.

Length 2.3–2.7 mm, widely oval. Eyes extremely reduced in size, dorsally eyes divided by distance of ca. 20 × the dorsal width of one eye. Elytra explanate laterally. Mesoventral plate subpentagonal, with strong marginal bead. Profemora without hydrofuge dense pubescence, only with sparsely arranged setae. Parameres gradually narrowing towards apex, rounded at apex. Median lobe a little longer than parameres, gradually narrowing from base, then abruptly wide and swollen subapically.

**Description.** *Habitus* (Figs 4–6). Body widely oval in dorsal view, moderately convex in lateral view. Length 2.3–2.7 mm (holotype: 2.5 mm), width 1.5–1.9 mm (holotype: 1.8 mm). *Coloration*. Dorsum of head, pronotum and elytron black, with lateral margins of pronotum and elytron, and anterior margin of head reddish yellow (Figs 4, 6). Maxillary palpi and antennae yellow-brown. Venter black, tarsi and mouthparts reddish yellow. *Head*. Clypeus with sparse, moderately coarse punctures, interstices smooth; anterior and lateral marginal portions of clypeus with clear microsculpture. Frons with punctation slightly denser and coarser than on clypeus. Eyes extremely reduced in size, dorsally eyes divided by distance of ca. 20 × the dorsal width of one eye, ventral portion absent. Labrum partly exposed in front of clypeus. Surface of mentum without microsculpture. *Thorax*. Pronotum with punctation consisting of punctures similar to those on frons but more or less sparser; surface between punctures smooth, without microsculpture; posterolateral corners acute, slightly projecting posteriad. Elytra explanate laterally, interval punctation consisting of punctures only slightly coarser than on pronotum; epipleura very wide. Mesoventral plate subpentagonal, ca. 1.2 × as long as wide, with strong marginal bead. Metaventrite short, metaventral process ca. of the same width throughout. Metanepisternum without anterolateral tooth. Profemora without hydrofuge dense pubescence, only with sparsely arranged setae. *Male genitalia.* Parameres ca. 1.9 × as long as phallobase, rather wide subapically, gradually narrowing towards apex, rounded at apex. Phallobase wide, posteriorly bearing long nearly symmetrical manubrium. Median lobe a little longer than parameres, gradually narrowing from base, then abruptly wide and swollen subapically; gonopore situated in apical 0.25 of median lobe.

#### Differential diagnosis.

See Table 1.

#### Etymology.

We dedicate the species to Dr. Li-zhen Li, an entomologist at Shanghai Normal University, as thanks for the donation of specimens collected by him.

#### Biology.

All known specimens were collected from terrestrial habitats. Holotype and nine paratypes were collected from mixed leaf litter in beech forest, and two specimens from dead wood with polypores in logging field.

#### Distribution.

China (Guangxi Province).

### Elocomosta nigra

Taxon classificationAnimaliaColeopteraHydrophilidae

Hansen, 1989

Figs 1–3 , 8 , 9 , 11 , 13 , 15–16 , 18 , 21–22 , 24 , 26 , 28 , 32

Elocomosta nigra Hansen, 1989: 255.

#### Type material examined.

Paratype. 1 female (NMPC): SARAWAK / Semengoh For. / Reserve. 11 mi. / SW Kuching / 1–4.vi.1968 / R. W. Taylor // PARATYPE / Elocomosta / nigra / M. Hansen.

#### Additional material examined.

1 female (NMPC): BORNEO: Sarawak / Kuching, Santubong / 26.3.1990 / leg. A. RIEDEL.

#### Redescription.

*Habitus*. Body widely oval in dorsal view, moderately convex in lateral view. Length 2.5–2.9 mm, width 1.7–1.9 mm. *Coloration*. Dorsum of head, pronotum and elytron uniformly black, margins only very indistinctly and narrowly paler. Maxillary palpi and antennae yellow-brown. Venter black to dark brown, tarsi and mouthparts reddish to reddish yellow. *Head*. Clypeus with sparse and very fine punctures, interstices smooth; anterior and lateral marginal portions of clypeus without microsculpture. Frons with punctation slightly coarser than on clypeus. Eyes well developed, strongly constricted laterally by anterior and posterior projections of frons; dorsal portion large, dorsally eyes divided by distance of 4.4 × the dorsal width of one eye, ventral portion small. Labrum nearly completely concealed by clypeus. Surface of mentum without microsculpture. *Thorax*. Pronotum with very fine and sparse punctation, punctures slightly smaller than on frons; surface between punctures smooth, without microsculpture; posterolateral corners rectangular, not projecting posteriad. Elytra not explanate laterally, interval punctation consisting of punctures coarser than on pronotum; epipleura moderately wide. Mesoventral plate circular, ca. as long as wide, with distinct but narrow marginal bead. Metaventrite short, metaventral process wide at base, narrowing anteriad. Metanepisternum with anterolateral tooth. Profemora with hydrofuge dense pubescence in basal 0.8. *Male genitalia*. Parameres ca. 1.4 × as long as phallobase, narrowly parallel-sided subapically, pointed at apex. Phallobase wide and long, posteriorly without manubrium. Median lobe a little longer than parameres, but with basal portion deeply inserted into phallobase, nearly parallel-sided below gonopore, apically abruptly narrowed into pointed apex; gonopore situated subapically.

#### Biology.

Terrestrial species, part of type series was collected from rainforest leaf litter (Hansen 1989). No biology data are available on locality labels in the specimens examined by us.

#### Distribution.

Endemic to Borneo (Sarawak State, Malaysia).

**Figures 9–17.**
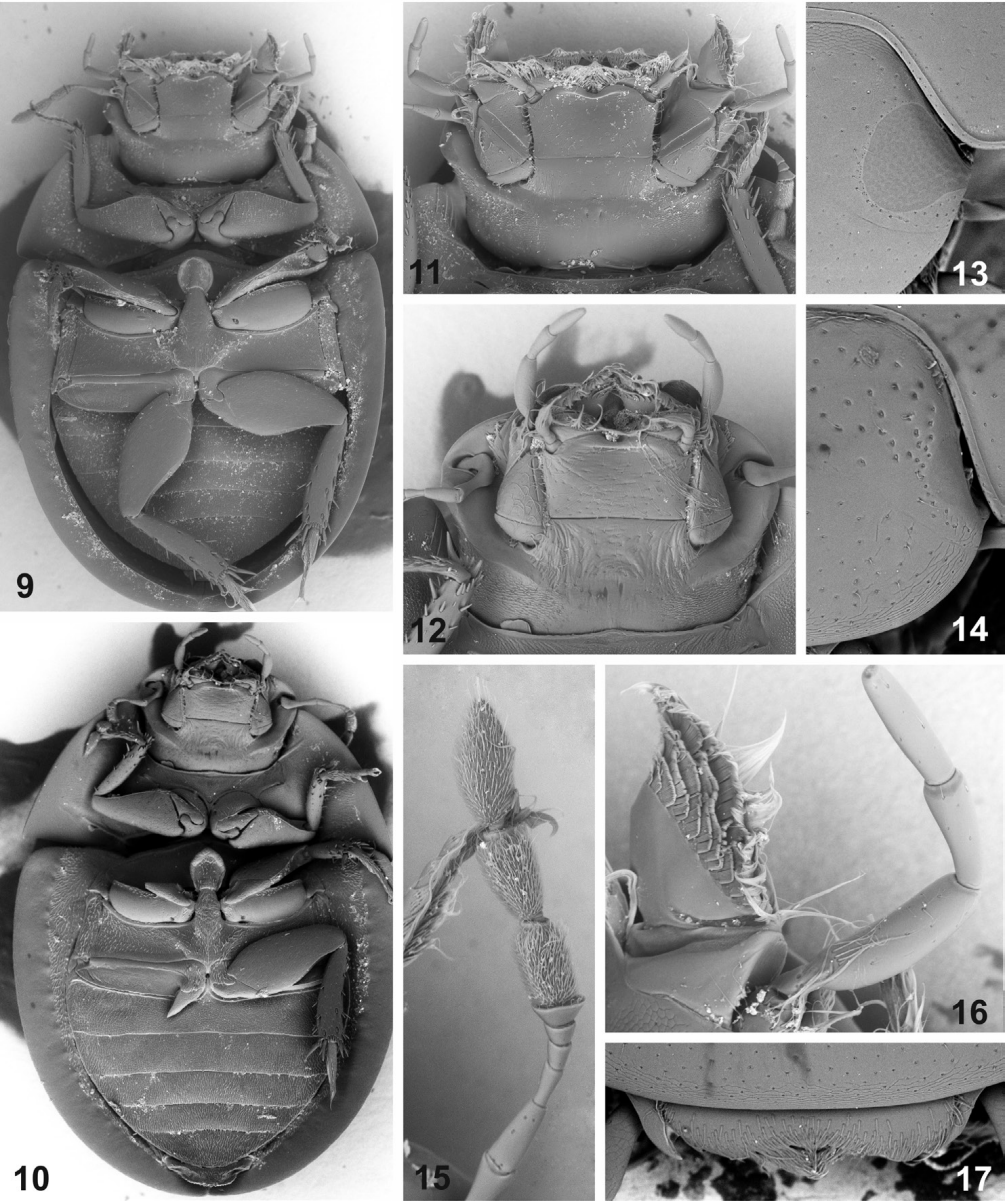
External morphology of *Elocomosta* species. **9–10** ventral morphology, general view (**9** *Elocomosta nigra* Hansen, 1989 **10** *Elocomosta lilizheni* sp. n.) **11–12** head, ventral view (**11** *Elocomosta nigra* **12** *Elocomosta lilizheni*) **13–14** detail of the ocular region of the head, dorsal view (**13** *Elocomosta nigra* **14** *Elocomosta lilizheni*) **15–16** head appendages of *Elocomosta nigra* (**15** antenna **16** maxillary palpus and galea) **17** anterior margin of head of *Elocomosta lilizheni* in dorsal view, showing largely exposed labrum.

**Figures 18–25.**
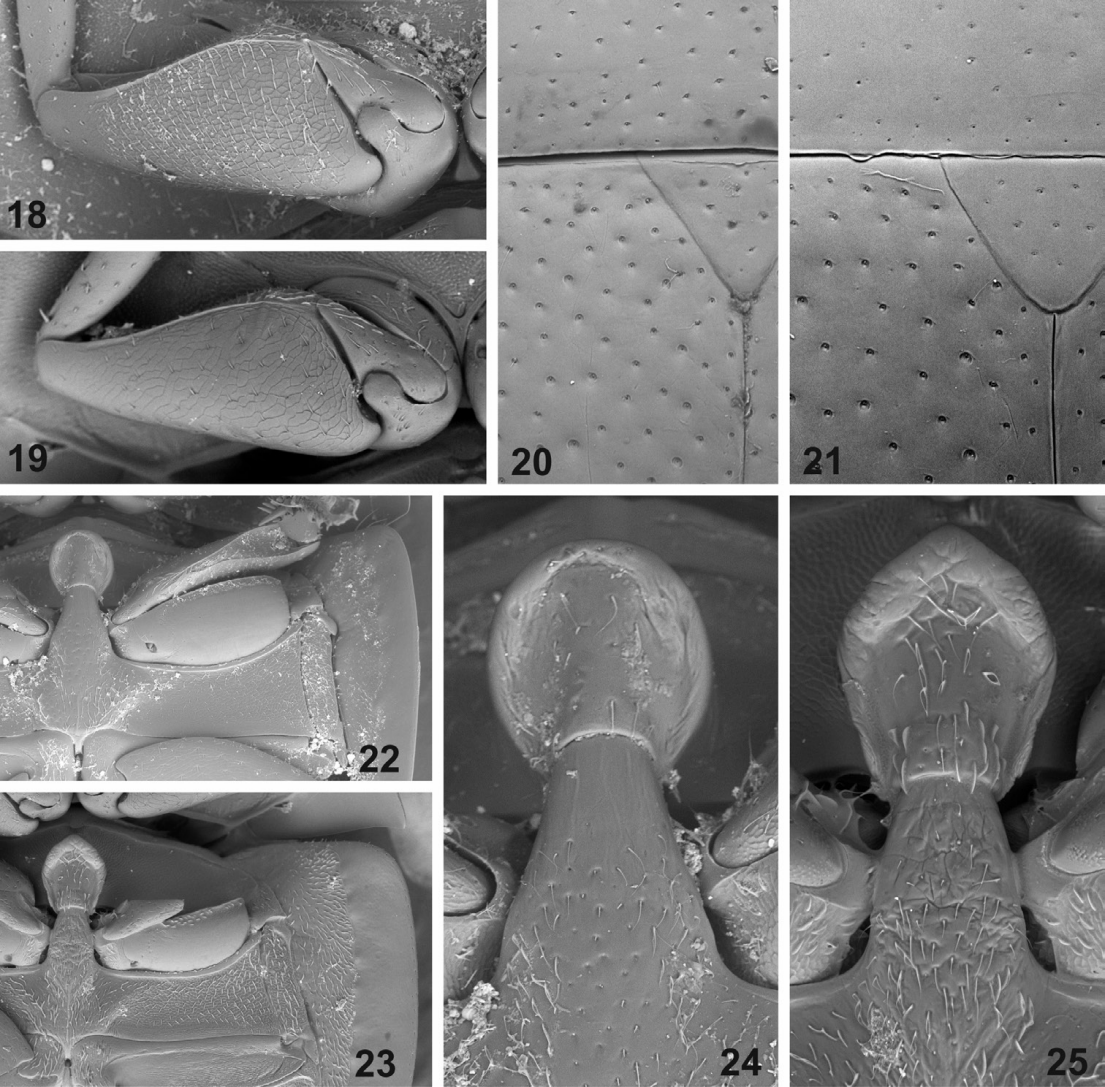
External morphology of *Elocomosta* species. **18–19** profemur (**18** *Elocomosta nigra* Hansen, 1989 **19** *Elocomosta lilizheni* sp. n.). **20–21** punctation of pronotum and elytral base (**20** *Elocomosta lilizheni* **21** *Elocomosta nigra*) **22–23** meso- and metaventral morphology (**22** *Elocomosta nigra* **23** *Elocomosta lilizheni*) **24–25** details of mesoventral plate and metaventral process (**24** *Elocomosta nigra* **25** *Elocomosta lilizheni*).

**Figures 26–33.**
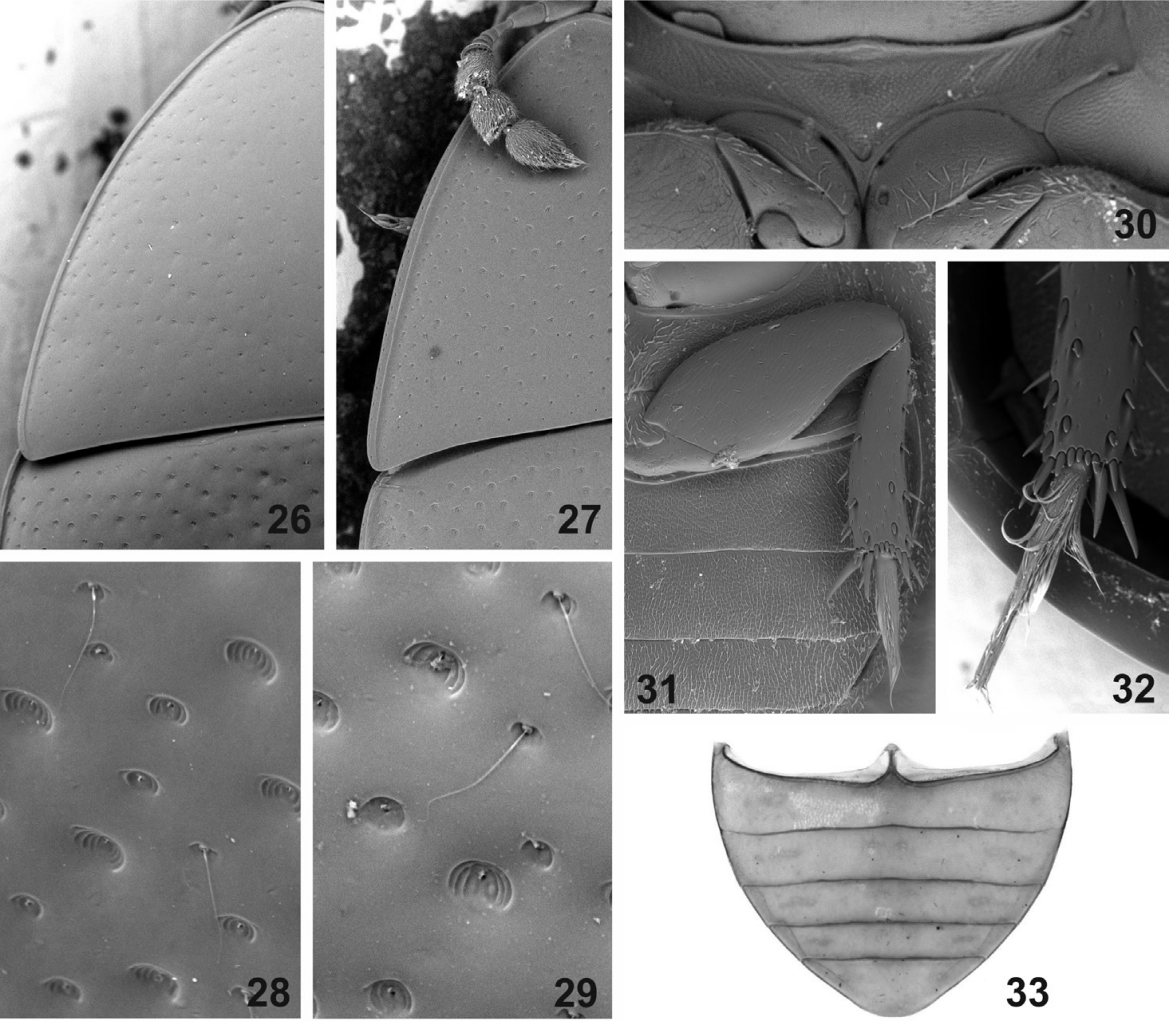
External morphology of *Elocomosta* species. **26–27** lateral portion of pronotum (**26** *Elocomosta nigra* Hansen, 1989 **27** *Elocomosta lilizheni* sp. n.). **28–29** details of elytral punctation and trichobothria (**28** *Elocomosta nigra* **29** *Elocomosta lilizheni*) **30** prosternum of *Elocomosta lilizheni* **31–32** posterior leg (**31** *Elocomosta lilizheni* **32** *Elocomosta nigra*) **33** abdomen of *Elocomosta lilizheni*.

## Discussion

The new species of *Elocomosta* is very unusual in Hydrophilidae due to its extremely reduced eyes. The examination using the scanning electron microscope revealed that the eye is not reduced completely, as it may appear from an observation under a stereomicroscope, but that the eye is extremely reduced in size, with only few ommatidia recognizable. Interestingly, it seems that not only the eye itself is reduced in size, but that the whole lateral portion of the head is reduced, which is best seen when the relative widths of the heads are compared between *Elocomosta nigra* and *Elocomosta lilizheni* sp. n. (compare Figs 3 and 6). This is unique among known Hydrophilidae, as in all known cases of eye reduction, the shape of the head remained unaffected or nearly so.

The reduction of eye size is commonly observed in the Sphaeridiinae, probably in relationship with the cryptic leaf-litter life style of the majority of the members of the subfamily. Reduced eye size was observed for example in some species of the megasternine genera *Motonerus* Hansen and *Oosternum* Sharp (Fikáček and Short 2006, Fikáček and Hebauer 2009) and seems to be frequently correlated with the reduction of the hind wings: in both aforementioned genera reduced eyes were observed in micropterous or apterous species. In case of *Elocomosta*, both *Elocomosta nigra* with well-developed eyes and *Elocomosta lilizheni* sp. n. with extremely reduced eyes are apterous. Moreover, the extent of eye reduction observed in *Elocomosta lilizheni* is not comparable to any other known member of the Sphaeridiinae, which may indicate not only flightlessness but also some other reasons which may be responsible for the eye reduction; a specialized lifestyle would be a possible candidate. For example, the loss of eyes was recently discovered in larvae of the myrmecophilous genus *Sphaerocetum* Fikáček which live inside of the nest of the *Crematogaster + Camponotus* ants, likely in its pupal and larval chambers (Fikáček et al. 2015). However, the collecting circumstances of *Elocomosta lilizheni* sp. n. do not conclusively indicate any highly specialized lifestyle nor an association with ants.

## Supplementary Material

XML Treatment for Elocomosta

XML Treatment for Elocomosta lilizheni

XML Treatment for Elocomosta nigra
